# Mesenchymal Stromal Cells Induce Podocyte Protection in the Puromycin Injury Model

**DOI:** 10.1038/s41598-019-55284-7

**Published:** 2019-12-20

**Authors:** Felipe Mateus Ornellas, Rodrigo J. Ramalho, Camilla Fanelli, Margoth Ramos Garnica, Denise M. A. C. Malheiros, Sabrina Vargas Martini, Marcelo Marcos Morales, Irene L. Noronha

**Affiliations:** 1 Laboratory of Cellular and Molecular Physiology, Institute of Biophysics Carlos Chagas Filho, Federal University of Rio de Janeiro, Rio de Janeiro, Brazil; 2 Laboratory of Cellular, Genetic, and Molecular Nephrology, Renal Division, University of São Paulo, São Paulo, Brazil

**Keywords:** Chronic kidney disease, Glomerular diseases

## Abstract

Podocytes are specialized cells with a limited capacity for cell division that do not regenerate in response to injury and loss. Insults that compromise the integrity of podocytes promote proteinuria and progressive renal disease. The aim of this study was to evaluate the potential renoprotective and regenerative effects of mesenchymal stromal cells (mSC) in a severe form of the podocyte injury model induced by intraperitoneal administration of puromycin, aggravated by unilateral nephrectomy. Bone derived mSC were isolated and characterized according to flow cytometry analyses and to their capacity to differentiate into mesenchymal lineages. Wistar rats were divided into three groups: Control, PAN, and PAN+ mSC, consisting of PAN rats treated with 2 × 10^5^ mSC. PAN rats developed heavy proteinuria, hypertension, glomerulosclerosis and significant effacement of the foot process. After 60 days, PAN rats treated with mSC presented a significant amelioration of all these abnormalities. In addition, mSC treatment recovered WT1 expression, improved nephrin, podocin, synaptopodin, podocalyxin, and VEGF expression, and downregulated proinflammatory Th1 cytokines in the kidney with a shift towards regulatory Th2 cytokines. In conclusion, mSC administration induced protection of podocytes in this experimental PAN model, providing new perspectives for the treatment of renal diseases associated with podocyte damage.

## Introduction

Podocytopathies are the most common group of glomerular diseases in which proteinuria is attributed to damage or dysfunction of podocytes^1^. The spectrum of podocytopathies includes minimal change disease (MCD), focal segmental glomerulosclerosis (FSGS), diffuse mesangial sclerosis, and collapsing glomerulopathy^2^. Podocytes are highly specialized cells, which are considered to be key cells for maintenance of the glomerular filtration barrier that prevents protein loss into the urine^3^. The magnitude of proteinuria is widely recognized as a marker of glomerulopathy and a risk factor for chronic kidney disease (CKD) progression^4^.

The puromycin aminonucleoside nephrosis (PAN) is a well-established rodent model that targets podocytes to cause damage with subsequent proteinuria, similar to human MCD^5^. Loss of podocytes after injury is considered a key factor in the pathogenesis of progressive glomerulosclerosis^6^. FSGS lesions can be obtained with cumulative PAN doses^7^, characterized by albuminuria, sclerotic lesions that are typically segmental, effacement of the podocyte foot processes and a reduction of podocyte markers such as Wilms’ tumor suppressor 1 (WT1) and nephrin. To induce more pronounced glomerular lesions, unilateral nephrectomy (UniNx) can be associated in this model^8^.

Owing to their terminal differentiation nature, podocytes have a limited capacity to undergo cell division and, thus, do not regenerate in response to injury, depletion or aging^9,10^. Excessive podocyte loss predisposes to glomerular disorders and subsequent chronic kidney disease (CKD). In this context, cell-based therapy due to extensive regenerative properties and paracrine effects has emerged as a potential strategy for the treatment of kidney diseases and could be of particular relevance in this setting. In fact, previous experimental studies have shown that cell therapy with mesenchymal stromal cells (mSC) administration promotes renoprotection, improving renal function and ameliorating the pathological changes^11–18^. Nevertheless, the possible role of mSC in podocyte injury has not yet been elucidated.

Thus, in the present study, we developed a severe podocyte injury model in Wistar rats, characterized by cumulative doses of the puromycin aminonucleoside and UniNx to mimic FSGS podocytopathy in humans, and we analyzed the possible renoprotective effects of mSC therapy on clinical and morphological parameters, as well as the possible modulatory effects of cytokines and vascular endothelial factor (VEGF).

## Results

### mSC therapy reduces proteinuria and albuminuria in the podocytopathy model

**Figure 1:**
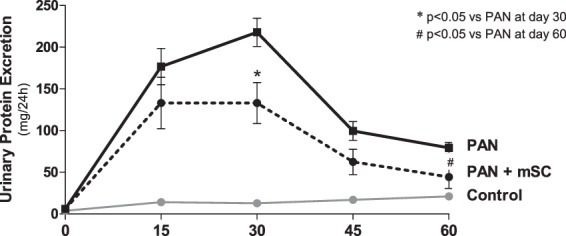
Urinary protein excretion analyzed on days 0, 15, 30, 45 and 60 in the different groups (Control: control animals submitted to UniNx; PAN: UniNx animals receiving PAN; PAN+ mSC: UniNx PAN animals treated with mSC).

PAN administration in Wistar rats subjected to UniNx (PAN group) induced severe proteinuria as early as day 15, with a peak on day 30 (Fig. 1), which was significantly greater than in the Control group. Subcapsular administration of mSC significantly reduced the 24 h urinary protein excretion and albuminuria at days 30 and 60, compared to the PAN group (Fig. 1, Table 1).

**Table 1:** Body weight, blood pressure, and albuminuria on days 0, 30 and 60 in the different groups (Control: control animals submitted to UniNx; PAN: UniNx animals receiving PAN; PAN+ mSC: UniNx PAN animals treated with mSC).

	Body Weight (g)			Blood Pressure (mmHg)			Albuminuria (mg/24 h)		
	0	Day 30	Day 60	0	Day 30	Day 60	0	Day 30	Day 60
Control	211 ± 5	369 ± 9*	456 ± 12*	127 ± 1	125 ± 2	123 ± 4	1.5 ± 0.5	1.5 ± 0.7	1.3 ± 0.3
PAN	209 ± 4	249 ± 15^#^	340 ± 17^‡^	126 ± 1	151 ± 5^#.‡^	167 ± 2^†,‡^	1.9 ± 0.9	242 ± 61^#^	205 ± 38^†^
PAN+ mSC	210 ± 5	249 ± 16^#^	376 ± 16^§^	127 ± 2	154 ± 3*^,#,§^	143 ± 6^†,§,¶^	1.0 ± 0.5	149 ± 27^#^	106 ± 22^†,¶^

*p < 0.05 vs. Control Day 0.

^#^p < 0.05 vs Control Day 30.

^†^p < 0.05 vs. Control Day 60.

^**‡**^p < 0.05 vs PAN Day 0.

^§^p < 0.05 vs PAN+ mSC Day 0.

^¶^p < 0.05 vs PAN Day 60.

Kidney injury developed in the PAN group was accompanied by high blood pressure levels, which were detected as early as day 30. Interestingly in the PAN+ mSC group, mSC promoted a significant decrease in blood pressure levels at day 60 in comparison to the PAN group (Table 1). Treatment with mSC in PAN animals had no effect on body weight (Table 2) or renal function, as analyzed based on the BUN and serum creatinine levels (Supplementary Table 1).

**Table 2:** Glomerular and interstitial inflammation was analyzed by immunohistochemical expression of ED1 (macrophages), CD43 (lymphocytes), and PCNA (proliferative activity) in the kidney cortical tissue in the different groups at days 30 and 60.

	ED1 (macrophages) (cells/mm^2^)				CD43 (lymphocytes) (cells/mm^2^)				PCNA (proliferative activity) (cells/mm^2^)			
	Glomerular		Tubulointerstitial		Glomerular		Tubulointerstitial		Glomerular		Tubulointerstitial	
	Day 30	Day 60	Day 30	Day 60	Day 30	Day 60	Day 30	Day 60	Day 30	Day 60	Day 30	Day 60
Control	27 ± 4	27 ± 4	28 ± 5	32 ± 4	3 ± 1	3 ± 1	65 ± 7	65 ± 7	18 ± 5	29 ± 3	28 ± 6	53 ± 1
PAN	251 ± 17*	166 ± 31*	463 ± 78*	292 ± 41*	4 ± 1	3 ± 1	78 ± 8	243 ± 17*	63 ± 3*	56 ± 8*	366 ± 7*	290 ± 29*
PAN+ mSC	154 ± 56*	130 ± 43*	173 ± 57*	146 ± 52*	3 ± 1	3 ± 1	62 ± 7	159 ± 30*	53 ± 3*	45 ± 6	184 ± 31*	156 ± 26*^#^

*p < 0.05 vs. Control Day 0.

#p < 0.05 vs Control Day 30.

### Effect of mSC on kidney morphology in rats with podocytopathy

**Figure 2:**
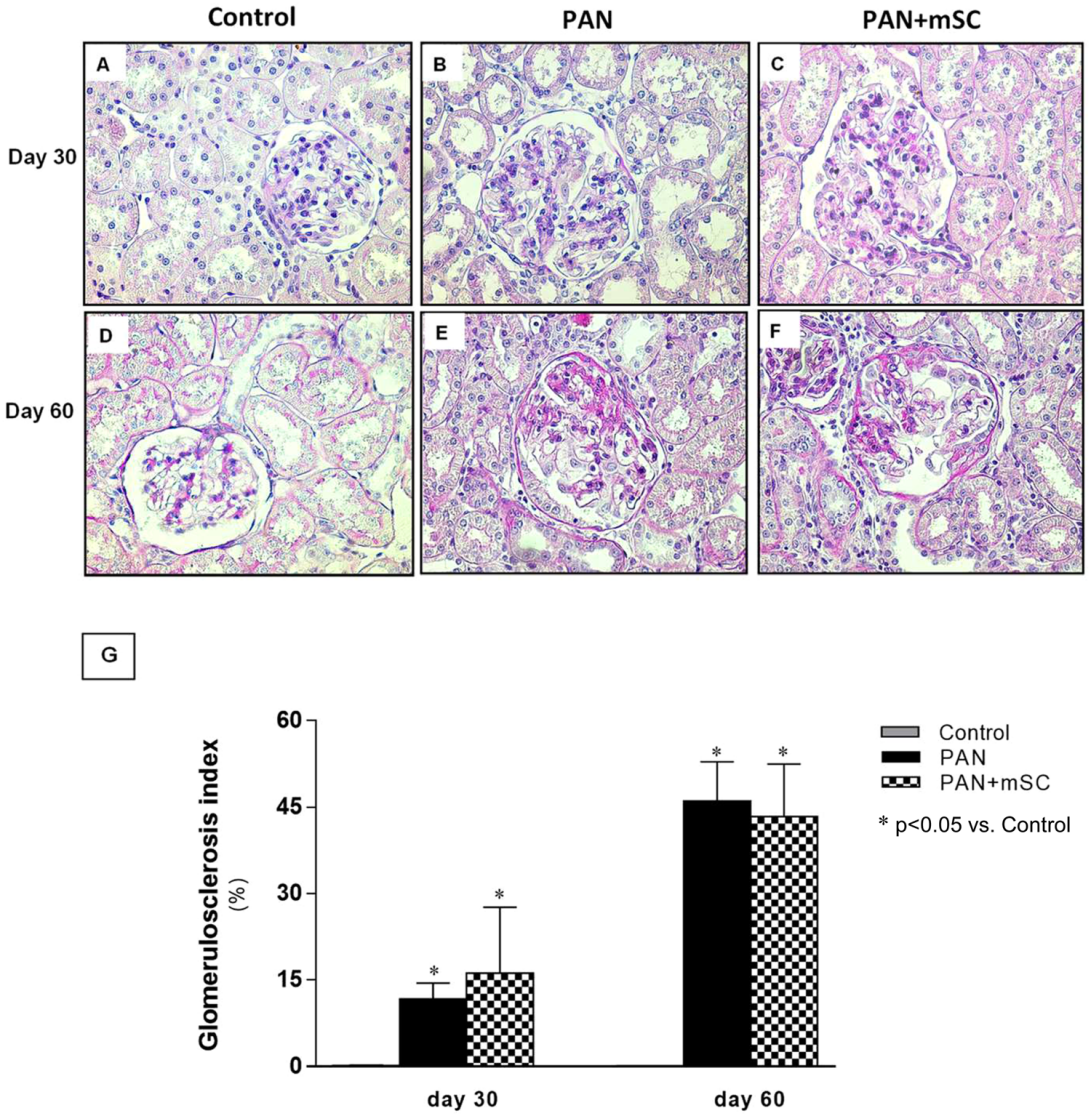
Glomerulosclerosis lesions evaluated at days 30 and 60 (400x magnification). The Control group at days 30 **(A)** and 60 **(D)** showed a conserved glomerular area. The PAN group presented marked glomerulosclerosis lesions at day 30 **(B)** that worsened significantly at day 60 **(E)**. Similar glomerulosclerosis lesions were observed in PAN+ mSC at days 30 **(C)** and 60 **(E)**. Histological scores for glomerulosclerosis in the different groups **(G)**.

The described podocyte injury model, characterized by PAN administration combined with UniNx (PAN group), induced glomerular lesions characterized by glomerulosclerosis, observed at day 30 and markedly at day 60 (Fig. 2). Analysis of histological scores for glomerulosclerosis using PAS staining did not reveal a difference between the PAN and PAN+ mSC groups. At 30 days, interstitial fibrosis was markedly increased in the PAN group. Treatment with mSC attenuated this parameter at day 30, but not at day 60 (Supplementary Fig. 1).

**Figure 3:**
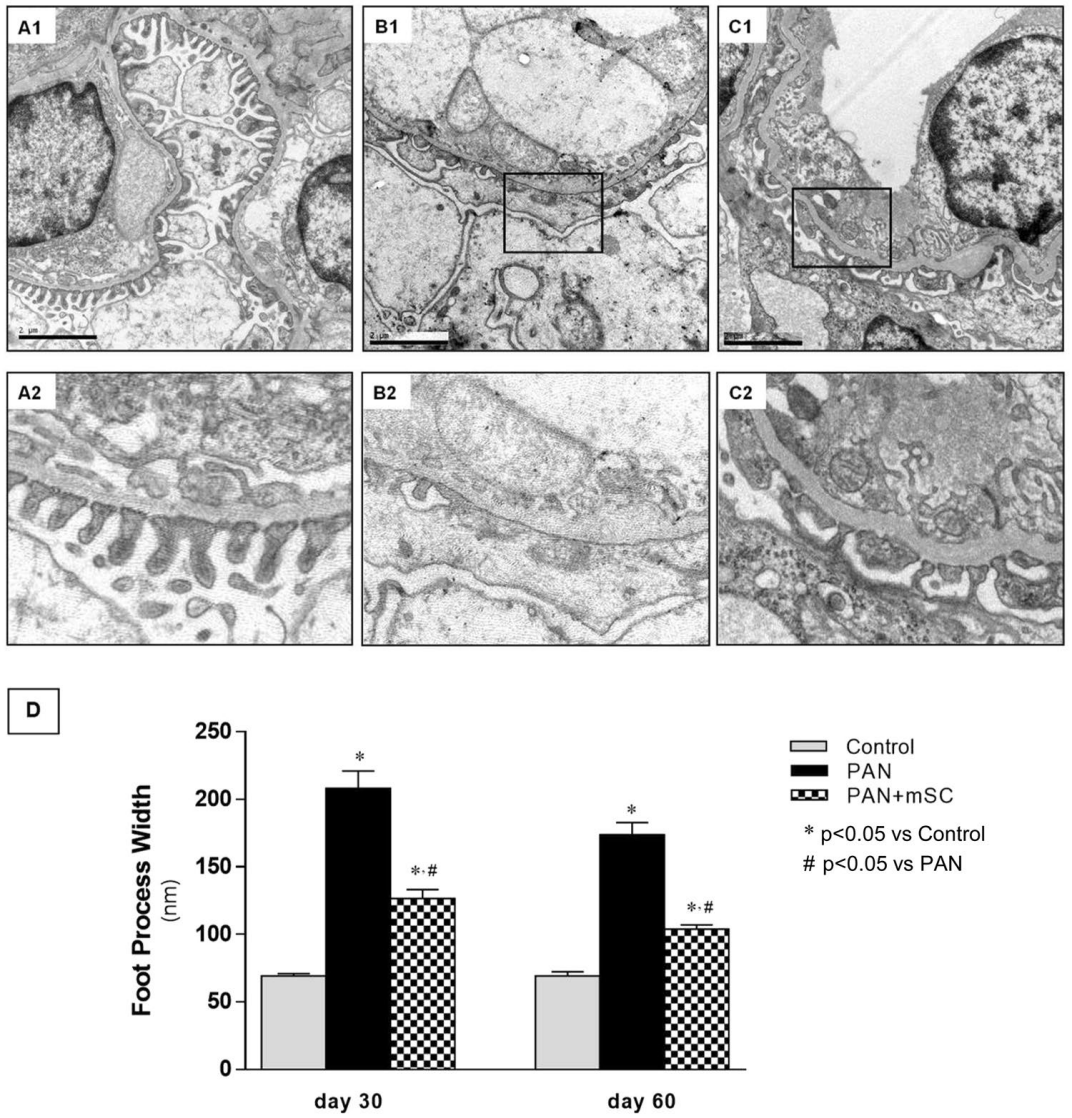
Electron microscopy analyses of kidney sections (15,000x magnification). Podocytes of control rats showed no abnormalities at day 60 **(A1**). The zoom indicates normal slit diaphragms **(A2**). In the PAN group, a loss of the normal slit diaphragm architecture was observed, which included crowding of the slit diaphragms and a reduced space between adjacent foot processes **(B1**) and **(B2**). The administration of mSC to PAN animals promoted lower effacement of the foot process **(C1**) and **(C2**). (**A1**, **B1** and **C1** 15000x magnification). **(D)** Quantification of the foot process width showed a significant improvement in the PAN+ mSC group compared with the PAN group at days 30 and 60.

Transmission electron microscopy analysis of the filtration barrier showed a normal podocyte morphology in the Control group. However, ultrastructural analyses demonstrated that PAN+ UniNx induced podocytopathy, characterized by significant effacement of the foot process, with almost completely absent slit-diaphragms, replaced by occluding junctions. Quantification performed at days 30 and 60 revealed notable ultrastructural changes induced by mSC and a significant decrease in effacement of the foot process compared with the PAN group (Fig. 3).

### mSC recovered WT1 expression in the glomeruli

**Figure 4:**
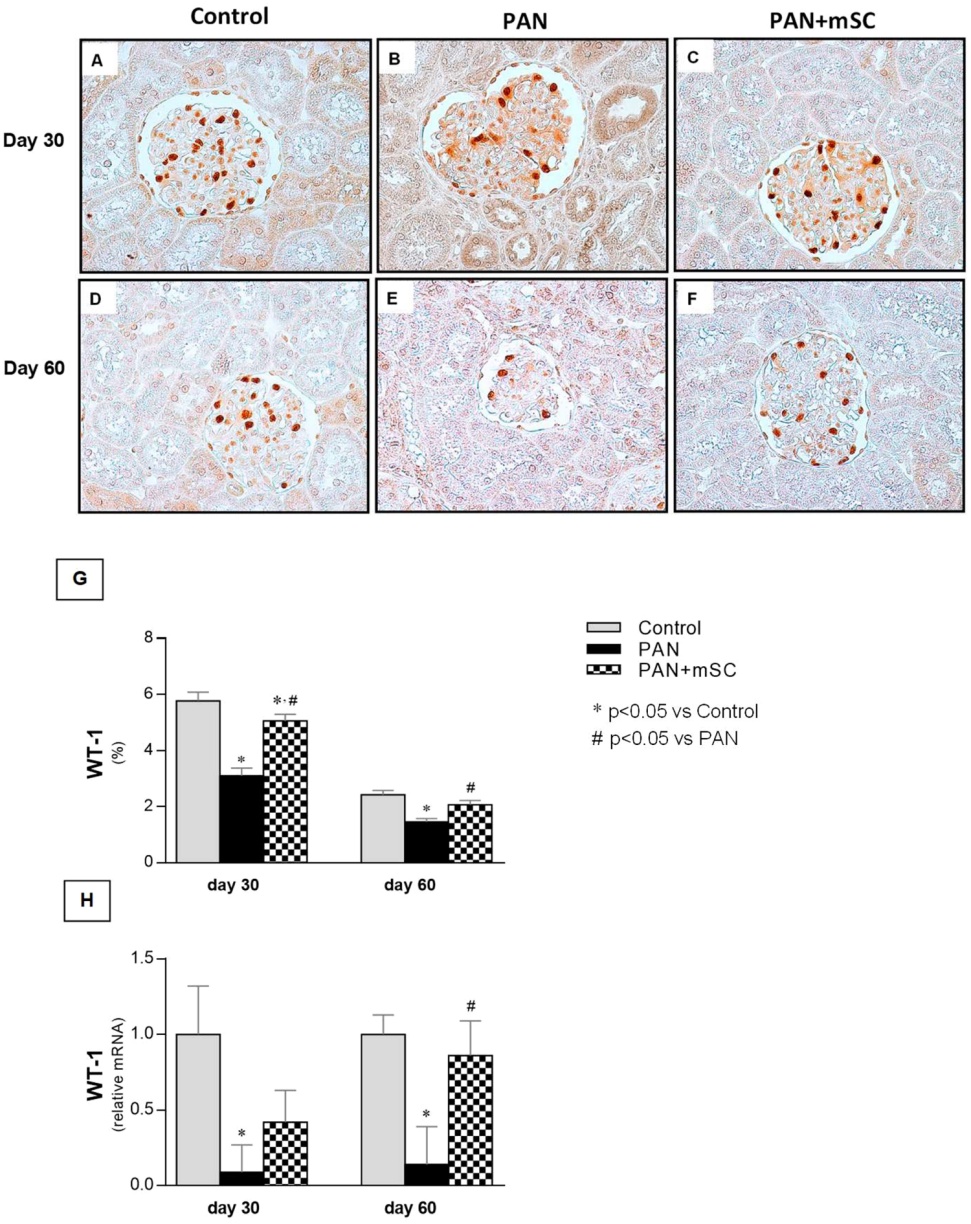
Expression of the podocyte marker WT1 analyzed by immunohistochemistry at days 30 and 60 in the different groups. Nuclear staining of WT1 was detected in the glomeruli in the control group **(A,D)**. A smaller number of WT1-positive cells was detected in the PAN group **(B,E)**. In the PAN+ mSC group, recovery of WT1 expression was observed at days 30 and 60 **(C,F)**. (400x magnification). Quantification of WT1 expression at the protein **(G)** and mRNA level in the different groups.

WT1 expression in the glomeruli was localized in the nuclei of podocytes. Podocyte loss induced by the PAN model in Wistar rats was confirmed by the significant decrease in WT1 expression, a podocyte marker, both at the mRNA and protein levels in the glomeruli (Fig. 4). mSC treatment led to progressive increases in WT1 expression in the PAN+ UniNx rat model. At day 60, mRNA levels of WT1 were significantly increased in the PAN+ mSC group compared with the PAN group, reaching values similar to the Control group. The analysis of WT1 expression, performed by immunohistochemistry, revealed a positive staining exclusively in glomerular cells. Notably, PAN resulted in a significant decrease in WT1 expression compared with the Control group at days 30 and 60. However, the administration of mSC in rats (PAN+ mSC group) recovered the WT1 expression in the PAN model.

### Loss of podocyte proteins was partially recovered by mSC

**Figure 5:**
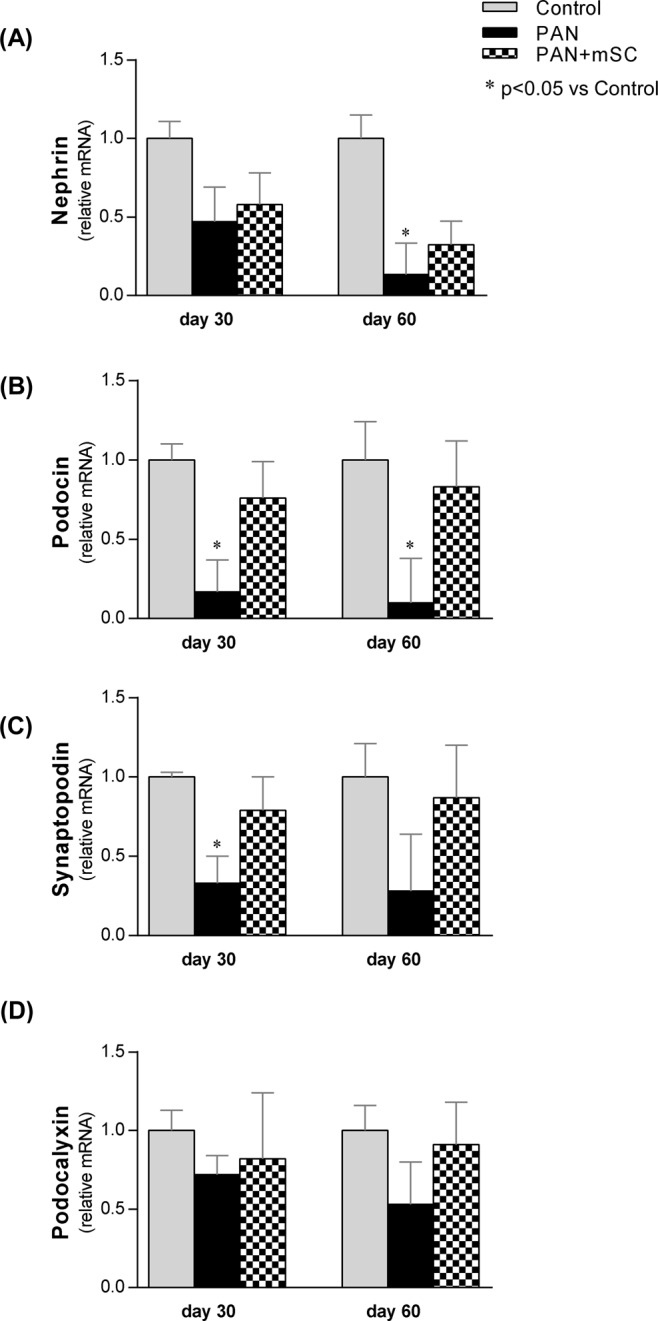
Comparative analysis of podocyte markers at days 30 and 60 in the different groups. The expression levels of nephrin **(A)**, podocin **(B)**, synaptopodin **(C)**, and podocalyxin **(D)** were analyzed in renal tissue by qRT-PCR.

The mRNA expression of nephrin and podocin, two proteins that are essential for the normal structure and function of the slit-diaphragm, was investigated. As shown in Fig. 5, nephrin and podocin mRNA levels were significantly decreased in the PAN group compared with the Control group. Treatment of PAN rats with mSC partially recovered the nephrin and podocin mRNA levels at days 30 and 60. The mRNA expression of synaptopodin (an actin cytoskeletal protein) and podocalyxin (an integral membrane protein) was also analyzed (Fig. 5). The PAN group presented a significantly lower mRNA expression of synaptopodin compared with the Control group at day 30. The synaptopodin mRNA level increased in the PAN+ mSC group, although no significance was reached. Podocalyxin expression did not change during the experimental period. The detailed results are shown in Supplementary Table 2.

**Figure 6:**
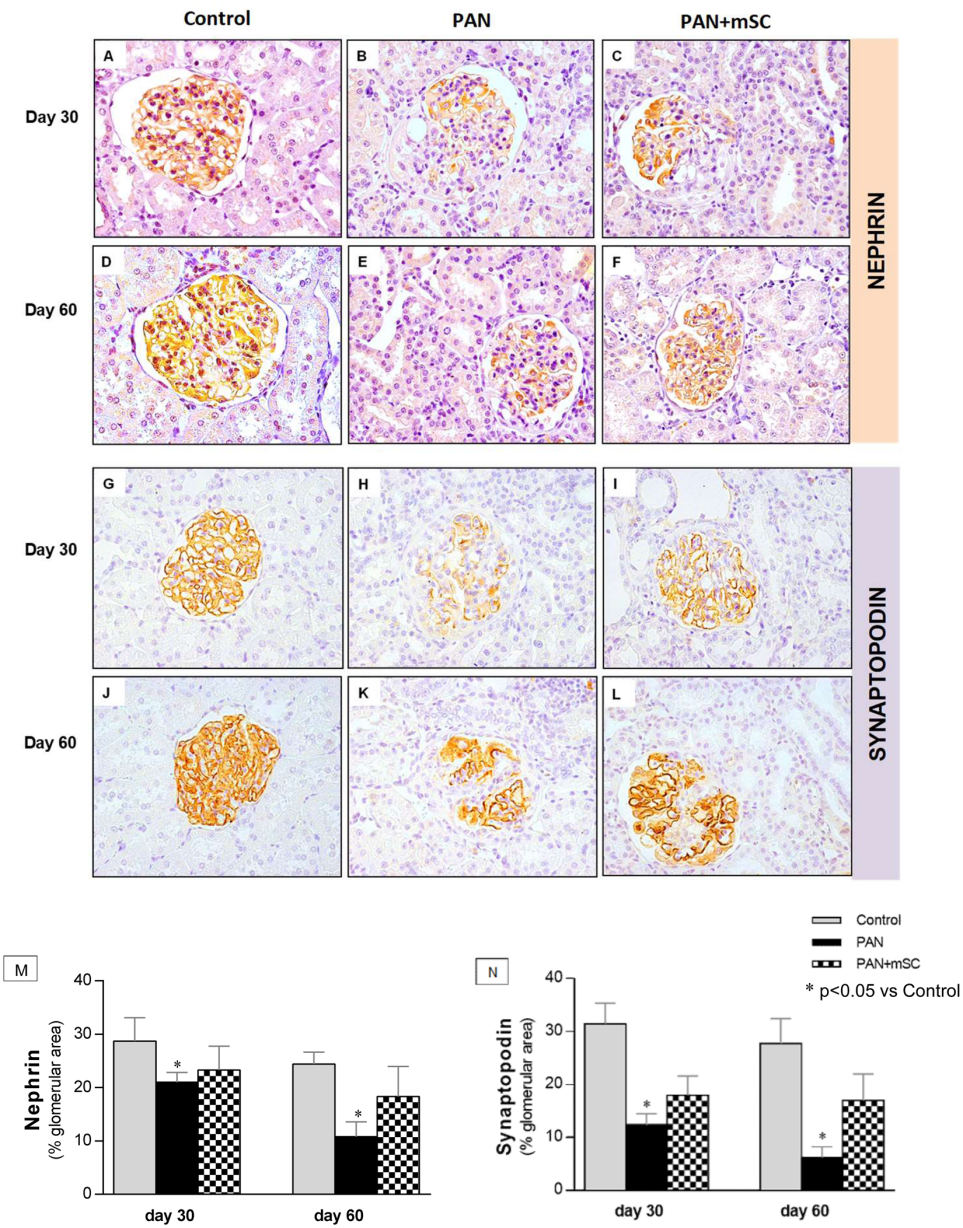
Glomerular expression of nephrin and synaptopodin was analyzed by immunohistochemistry at days 30 and 60 in the different groups. Normal expression of these podocyte markers was observed in the illustrative microphotographs (400x magnification) of the Control group **(A,D,G,J)**. The PAN group **(B,E)** showed lower nephrin expression than the Control group at days 30 and 60. The mSC administration improved nephrin expression **(C,F)** compared with the PAN group, particularly at day 60. Similarly, synaptopodin expression was lower in PAN animals compared with the Control group at days 30 and 60 **(H,K)**. Synaptopodin expression was upregulated by mSC **(I,L)**. Results of the quantitative histomorphometry by means of an image analysis system is shown in graphs (**M,N**).

Immunohistochemistry experiments for nephrin and synaptopodin confirmed the data obtained with qPCR analysis showing that the expression of these proteins was significantly low in the glomeruli of PAN group, with a partial recovery by the mSC treatment (Fig. 6).

### Inflammatory mechanisms underlie the podocyte injury model induced by PAN and are ameliorated by mSC

In order to investigate the possible role of mSC on inflammatory mechanisms in this podocyte injury model, the expression of inflammatory cytokines and the inflammatory cellular infiltrate in kidney tissue were analyzed.

**Figure 7:**
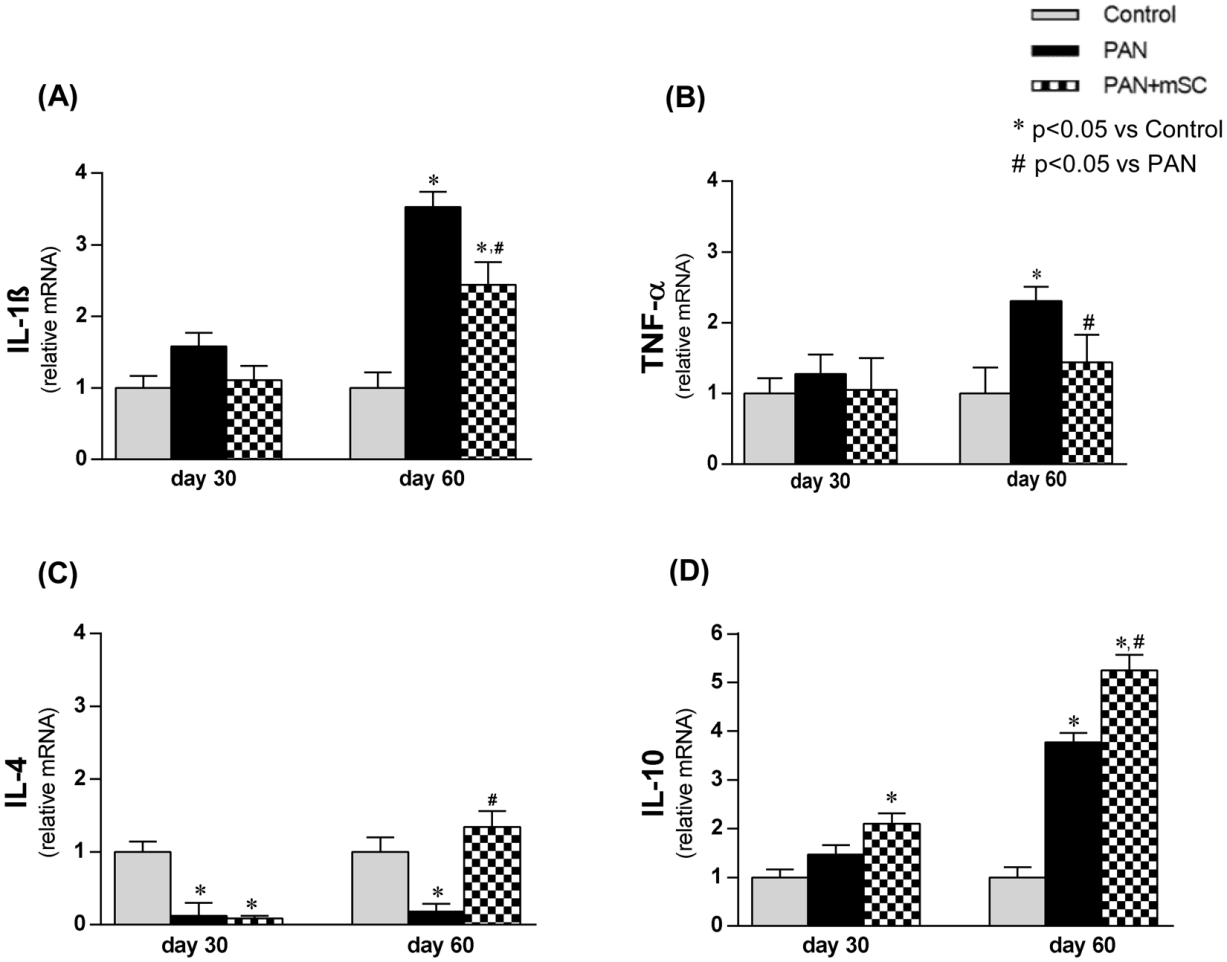
Comparative analysis of inflammatory cytokines after 30 and 60 days in the different groups. The expression levels of IL-1β **(A)**, TNF-α **(B)**, IL-4 **(C)** and IL-10 **(D)** were analyzed in renal tissue by real time RT-PCR.

The relative expression levels of IL-1β, TNF-α, IL-4 and IL-10 were analyzed by real-time RT-PCR to investigate cytokine-mediated inflammatory mechanisms (Fig. 7). A significant increase of the pro-inflammatorycytokines IL-1β and TNF-α was observed on day 60. However, a significant reduction of these cytokines in the PAN+ mSC group compared with the PAN group at day 60 was observed. Furthermore, mSC induced a Th2 immune response profile in this model, as evidenced by a significant increase in IL-4 and IL-10 gene expression in the renal tissue in the PAN+ mSC group compared with the PAN group. The detailed results are shown in Supplementary Table 3.

Macrophage and lymphocyte infiltration, as well as cell proliferative activity, were analyzed in both glomerular and tubulointerstitial compartments of kidney samples by means of immunohistochemistry. The number of macrophages was markedly increased in PAN animals both at days 30 and 60 when compared with the Control groups, particularly in the tubulointerstitial compartment. Lymphocyte infiltration and proliferative activity were also observed in this model, characterizing an inflammatory process associated with this model. Treatment with mSC reduced macrophage and lymphocyte infiltration, as well as cell proliferation (Table 2). In parallel, PAN animals showed a significantly higher number of apoptotic cells at both days 30 and 60 when compared with controls. mSC treatment promoted a significantly lower occurrence of apoptosis in PAN rats after 60 days. The paucity of apoptotic cells detected in the glomeruli of rats subjected to the PAN model is noteworthy, possibly because these analyses were performed at later stages after the PAN exposure.

### mSC induced a significant increase in PDGF expression in the kidney in the PAN model

**Figure 8:**
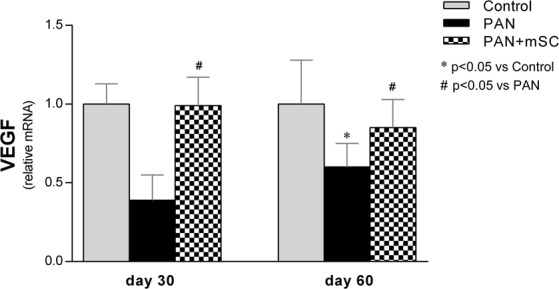
Comparative analysis of VEGF after 30 and 60 days in the different groups. The expression of VEGF was analyzed in renal tissue by real time RT-PCR.

To further evaluate the effect of mSC in this model, the relative expression of the growth factor VEGF was analyzed in renal tissue by real-time RT-PCR (Fig. 8). The relative expression of VEGF was significantly reduced in the PAN group compared with the Control group at day 60. The mSC treatment was able to significantly increase the mRNA levels of VEGF at day 60. The detailed results are shown in Supplementary Table 3.

## Discussion

In this study, we evaluated the potential renoprotective effects of mSC in the podocyte injury model induced by PAN in rats. The administration of one dose of PAN promoted a transient proteinuria, without significant glomerulosclerosis, resembling MCD in humans. In order to generate a more severe model of podocytopathy, in addition to receiving two doses of PAN, animals were subjected to unilateral nephrectomy (UniNx) simultaneously with the induction of podocyte damage. Indeed, the animals developed massive proteinuria as early as day 15, marked glomerulosclerosis and significant effacement of the foot process, which were observed until day 60, resembling the clinical situation of patients with podocytopathies^19^.

mSC were implanted underneath the kidney capsule to maximize the presence of these cells closest to the site of the lesion, aiming to facilitate the homing and engraftment of stem cells to the injured region. In our previous study, we have shown the trafficking of mSC to the renal cortex after subcapsular inoculation, providing an efficient delivery of these cells to the kidney. Five days after inoculation, the mSC were localized as infiltrating the glomeruli and renal interstitium, reaching the renal medulla 15 days later, and being detected up to 30 days using this delivery route^15^. Thus, the approach of inoculating mSC in the subcapsular area of the kidney may avoid systemic dispersion of these stem cells to other organs, mainly to the lungs and the liver, when using the intravenous route^14,20^.

The results of this study demonstrated that mSC administration in PAN rats promoted renoprotective effects, characterized by a significant reduction of blood pressure levels, proteinuria, albuminuria, and serum creatinine in PAN+ mSC rats. The effects of mSC in ameliorating blood pressure levels in the PAN model is of clinical interest, although the mechanisms are not clear. A reduction of blood pressure levels was also observed with mSC therapy in another model of experimental CKD, the 5/6 nephrectomy model, indicating a beneficial effect of mSC administration^15^. However, the most relevant effect of mSC administration in the podocyte injury model was the reduction of proteinuria and albuminuria levels. It is well known that proteinuria is a biomarker for kidney dysfunction and an important prognostic factor for the progression of kidney disease^4^. Our results are in agreement with previous reports that mSC administration results in decreased protein excretion in the 5/6 nephrectomy model^15^ and in the anti-Thy glomerulonephritis 1.1 model^21^, as well as in mice receiving one single dose of PAN, demonstrating a protective role of CTm in the reduction of proteinuria^22^. On the other hand, mSC administration in the adriamycin-induced nephropathy model did not modify the proteinuria^16,17^.

The possible renoprotective effects of mSC in decreasing the proteinuria observed in the present study are likely closely related to the recovery of WT1 expression and the amelioration of the functional and structural integrity of podocytes, particularly proteins associated with the slit diaphragm (nephrin and podocin) as well as cytoskeletal proteins (synaptopodin and podocalyxin). While the induction of podocyte injury led to significant alterations in molecular composition, characterized by decreased WT1 expression and the downregulation of nephrin, podocin, synaptopodin, and podocalyxin expression, the administration of mSC in this disease model has shown a protective role by inducing a significant upregulation of WT1 and partial recovery of nephrin, synaptopodin, podocin, and podocalyxin expression. Our data confirm previous findings regarding the protective effect of mSC on podocytes^17,22,23^.

Interestingly, in the model of progressive glomerulosclerosis induced by adriamycin, although mSC reduced podocyte depletion and preserved nephrin expression, they did not reduce proteinuria^16,17^. These contrasting results are not clear, but they may indicate that the normal function of podocytes was not reestablished due to a limited recovery of the podocyte slit diaphragm protein structure of the foot process. In these experiments, the number of mSC infused intravenously (2–3 × 10^6^) or intraperitoneally (9 × 10^6^) was significantly greater than in our protocol (versus 2 × 10^5^). However, despite the higher number or repeated infusions of mSC in adriamycin rats to provide a constant number of mSC in the kidney, the administration of mSC by intravenous tail-vein infusion or intraperitoneally may not have delivered the same amount of mSC into the kidney as the subcapsular inoculation strategy used in our study, which may account for the different results. Indeed, the number of stem cells in the kidney necessary to play a role in the renoprotective effects has not yet been established.

Although mSC administration to rats subjected to the podocyte injury model had no effect on the degree of glomerulosclerosis, analyses by electron microscopy evidenced ultrastructural improvement. In the animals exposed to the PAN model, characteristic lesions of podocytopathies were observed, with pronounced effacement and fusion of the podocyte foot process, consistent with the lower levels of proteinuria observed. Similar findings have been described in the PAN model developed in mice^17^.

The evaluation of cytokine expression in the kidney tissue in this study corroborated previous reports describing mSC-induced anti-inflammatory and immunomodulatory effects^24,25^. Sixty days after induction of the podocyte injury model, the proinflammatory environment observed in rat kidneys, characterized by marked increased expression of IL-1ß and TNF-α, was downregulated by the treatment of these animals with mSC. In parallel, a Th2 cytokine profile, characterized by a significant increase in IL-4 and IL-10 mRNA expression, was induced with mSC administration in this disease model. Previous *in vitro* experiments have demonstrated the ability of mSC to directly promote the production of IL-10 in alloantigen activation^26^. Taken together, the present data showed that mSC promoted a shift from the pronounced proinflammatory Th1 towards an increase in regulatory Th2 cytokines.

Treatment of PAN rats with mSC also influenced renal interstitial inflammatory infiltrate and the cellular proliferative activity in the kidney. mSC-treated animals exhibited reduced interstitial macrophage infiltration and a significant reduction of tubulointerstitial PCNA-positive cells, indicating local anti-inflammatory effects of mSC administration in PAN rats. However, the limited proliferative activity observed in the glomerular compartment in the PAN model treated with mSC demonstrated a limited ability to induce podocyte proliferation, possibly due to the limited capacity of podocytes to undergo cell division and, thus, limiting a regenerative process in response to injury. Recent studies have shown that podocyte replacement may occur but is dependent on podocyte progenitors present in the kidney, such as a subpopulation of CD133+CD24+ cells localized within Bowman´s capsule^12^ or of renin lineage cells^27,28^.

Finally, the expression of VEGF in the kidney of the podocyte injury model and the effects of mSC therapy in this setting were also investigated. VEGF, one of the most important factors responsible for the integrity of podocytes, is strongly synthesized by podocytes and glomerular endothelial cells^29,30^. The concomitant expression of VEGF receptors in podocytes provides paracrine and autocrine effects of this growth factor, which are crucial for regulation, maintenance and increased podocyte survival^30,31^, possibly mediated by nephrin phosphorylation^32^. Studies performed in conditionally mouse podocytes have also shown the ability of VEGF to increase podocin expression, confirming an important role of VEGF in the regulation of slit diaphragm proteins^30^. In the present study, the decreased expression of VEGF observed in the PAN group, in parallel with the development of proteinuria and decreased expression of nephrin, podocin, synaptopodin and podocalyxin, may explain the important role of this growth factor in maintaining podocyte function and the glomerular filtration barrier.

In fact, the reduction of VEGF synthesis by podocytes in transgenic mice has been shown to induce proteinuria^33^. Inhibition of VEGF activity (either with anti-VEGF antibodies or with soluble VEGF receptors) has been shown to induce podocyte dysfunction with massive proteinuria in normal kidney in mice^34^ and in nephritic rats^35^. In clinical oncology, the use of anti-VEGF antibodies to treat patients with different types of cancer has been shown to induce proteinuria as an adverse event associated with these treatment protocols^36^.

The results of the present study showed that mSC administration induced a recovery of VEGF expression in the kidney, which was downregulated after induction of the podocyte injury model. Zoja and coworkers reported similar renoprotective effects of mSC in adriamycin-induced nephropathy^17^.

It is well known that mSC can secrete high levels of VEGF, among a variety of other growth factors^13,37^. In experimental models of acute kidney injury treated with mSC, VEGF is considered to be one of the major paracrine mediators inducing renoprotective effects^13^. Previous experiments examining the coculture of mSC with immortalized podocytes injured after adriamycin exposure, have shown beneficial effects of stem cell-derived VEGF on prosurvival pathways^17^. Thus, it seems that local VEGF release by mSC could have a role in promoting podocyte survival and the recovery of slit diaphragm proteins, thus ameliorating podocyte injury and proteinuria.

In summary, mSC therapy in this model was able to induce renal protection characterized by a reduction of albuminuria, proteinuria and blood pressure, associated with lower effacement of the foot process and increased gene expression of podocyte proteins and cellular expression of WT1. Downregulation of proinflammatory Th1 cytokines with a shift to an increase in regulatory Th2 cytokines, associated with increased VEGF expression in the kidney, were the probable mediators in the amelioration of podocyte injury. Our study supports the use of mSC with immunomodulatory properties as an alternative in the treatment of podocytopathies.

## Methods

### Animal model

All animal protocol studies were approved by the Ethics Committee for Analysis of Research Projects (CAPPesq 0585/06), and were performed in accordance with our institutional guidelines and with international regulations for manipulation and care of experimental animals. Male Wistar rats (180–230 g, 8–9 weeks old) were obtained from an established colony at the University of São Paulo, Brazil. They were maintained at a constant temperature (23 ± 2 °C) under a 12-h light/dark cycle and fed a standard rat diet (Labina, Purina Agribrands, Paulinia, SP, Brazil) and filtered water ad libitum. No more than 3 animals were kept per cage during the experiments.

### Experimental design

The PAN model was induced in male Wistar rats aggravated with UniNx to accelerate the onset of lesions with features of chronic disease. At day 0, the animals were anesthetized with ketamine-xylazine (100/20 mg/kg) intraperitoneally and subjected to a dorsal incision on the right side to perform UniNx. After UniNx surgery, the animals received PAN (150 mg/kg) intraperitoneally. After 15 days, a second dose of PAN was administered intraperitoneally. For the PBS or mSC inoculation, a dorsal incision was generated on the left side. A 5-mm incision was made in the capsule of the left kidney, a sterile glass capillary was placed under the kidney capsule and 2 × 10^5^ mSC (in 10 µl sterile PBS) were injected with the help of a micro-injector.

Fifty-one animals were divided randomly assigned into three groups: Control (rats submitted to unilateral nephrectomy – UniNx; n = 15), PAN (PAN administration in UniNx rats, treated with PBS under the kidney capsule; n = 18), and PAN+ mSC (PAN rats treated with 2 × 10^5^ mSC under the kidney capsule; n = 18). The groups were followed for 30 and 60 days, and euthanasia was performed by anesthesia with sodium pentobarbital 50 mg/kg intraperitoneally.

### Isolation, expansion, and characterization of rat mSC

Bone marrow cells were obtained from femurs and tibias of male Wistar rats (180–230 g). After isolation, 1 × 10^7^ bone marrow-derived cells were cultured (37 °C, 5% CO_2_) in T25 culture flasks (TPP, Schaffhausen, Switzerland) with Dulbecco’s Modified Eagle’s Medium (DMEM; Invitrogen, Carlsbad, CA, USA) containing 15 mM HEPES (Sigma, St. Louis, MO, USA), 10% inactivated fetal bovine serum (FBS; Invitrogen), 100 units/mL penicillin, and 100 mg/mL streptomycin antibiotic solution (Gibco, Carlsbad, MO, USA). After 2 weeks of culture, the medium was changed, and nonadherent cells were removed. Adherent cells reaching 80% confluence were passaged with 0.05% trypsin–ethylenediaminetetraacetic acid solution (Gibco) and then maintained in DMEM with 10% FBS (complete medium).

At the fourth passage, cells were characterized as mSC according to the International Society of Cellular Therapy Consensus, i.e., adherent to plastic under standard conditions, expressing some surface markers (CD29, CD44, CD90, and CD105) and lacking the expression of others (CD34, CD31, and CD45), and the capacity to differentiate into mesenchymal lineages under *in vitro* conditions. Cells were used at the 4^th^ passage according to flow cytometry analyses (FACScalibur cytometer equipped with a 488 nm argon laser (Becton Dickinson, San Diego, CA, USA) with CellQuest software). For this purpose, cells were labeled with isothiocyanate (FITC)-conjugated antibodies against CD31, CD29, and CD90, phycoerythrin (PE)-conjugated antibodies against CD34, CD44, and CD105, Pe-cy5.5-conjugated antibody against CD45, and FITC- or PE-conjugated nonspecific IgG (Caltag Laboratories, Carlsbad, CA, USA).

The potential of mSC to differentiate into mesenchymal lineages including osteoblasts, chondroblasts, and adipocytes under *in vitro* conditions was evaluated. Osteogenic differentiation was induced by culturing MSC for up to 3 weeks in DMEM 10% FBS and 15 mM HEPES (Sigma), supplemented with 10^−8^ M/L dexamethasone (Sigma), 5 μg/mL ascorbic acid 2-phosphate (Sigma), and 10 mM/L β-glycerolphosphate (Sigma). To observe calcium deposition, cultures were stained with Alizarin Red S (Nuclear, São Paulo, SP, Brazil). To induce chondrogenic differentiation, MSC were cultured in DMEM supplemented with 10 ng/ml transforming growth factor (TGF)-β1 (Sigma), 50 nM ascorbic acid 2-phosphate (Sigma), and 6.25 mg/ml insulin for 3 weeks. To confirm differentiation, cells were fixed with 4% paraformaldehyde in phosphatebuffered saline (PBS) for 1 hour at room temperature and stained with Alcian Blue pH 2.5. Adipogenesis differentiation was induced by culturing MSC for up 3 weeks in DMEM supplemented with 5 µg/ml insulin, 10^−6^ M dexamethasone, 0.5 µM isobutylmethylxanthine, and 50 µM indomethacin. The cells were then fixed with 10% formalin, and oil red O staining was used to visualize the accumulation of lipid droplets into the cell vacuoles.

### Clinical and biochemical parameters

Tail-cuff blood pressure was performed using the Kent RTBP2000 system and the Kent RTB001-R data acquisition system (Kent Scientific Corporation, Torrington, CT, USA), and body weight was measured in conscious animals at days 0, 30 and 60.

To analyze the level of 24 h urinary protein excretion (sulfosalicylic acid turbidity test) and urinary albumin excretion (ELISA technique, Bethyl Laboratories, Montgomery, AL, USA), rats were maintained in metabolic cages for 24 h before urine collection at days 0, 15, 30, 45 and 60. Blood urea nitrogen (BUN) was measured according to Crocker’s protocol (Celm, Barueri, Brazil), and serum creatinine was determined using a colorimetric assay (Labtest, Lagoa Santa, Brazil) at day 0, 30 and 60.

### Renal histology

One midcoronal section/animal of the left kidney was fixed in Dubosq-Brazil solution for 45 minutes and then post-fixed in buffered 10% formaldehyde solution, and 2- to 3-mm-thick sections were stained with periodic acid–Schiff (PAS) reagent and with the Masson Trichrome technique. The extent of glomerulosclerosis was evaluated by attributing a score to each glomerulus according to the extent of sclerotic lesions and it was expressed as the percentage of the total number of glomeruli examined. All morphometric evaluations were performed in a blinded manner by a single observer.

Glomerular volume was analyzed in PAS-stained renal slides through a point-counting technique, employing a 160-point grid, covering a total area of 70,000 mm^2^. Fifty consecutive glomerular tuft profiles of each studied animal were observed under 400x magnification, and the number of points falling within the tuft profiles were counted to estimate the mean glomerular area (GA). Glomerular tuft volume (GV) for each slide was then calculated as GV = [1.25 (GA)^1,5^] and expressed as “X 10^6^ μm^3^”.

The presence of tubular atrophy and tubular casts was analyzed in 25 consecutive microscopic fields of PAS-stained sections of each experimental group, under 200x magnification.

### Transmission electron microscopy procedure

Fresh kidneys were randomly selected and underwent primary fixation with 2% glutaraldehyde in sodium phosphate buffer. They were then post-fixed in 1% osmium tetroxide for 1 hour and dehydrated in ethanol and propylene oxide. The samples were further infiltrated with epoxy resin mixture. Ultra-thin sections were collected on copper grids, and the sections were stained using 10% uranyl acetate. A Jem-1010 electron microscope (Jeol Ltd, Tokyo, Japan) was used for image acquisition. To determine the average foot process width, 20 electron microscopy images from 10 animals in each group were examined at 15,000x magnification. In each image, the curved total length of the basement membrane (BML) was measured using Image-J. By counting the number slit diaphragms, the average foot process width (Wp) was calculated using the formula Wp = *π*/4 × Σ BML/Σ slits (45). The results are expressed in nanometers (nm).

### Immunohistochemistry

Immunohistochemical procedures were performed using 4-μm-thick, paraffin-embedded kidney sections using the following antibodies: WT1 (anti-human WT1 monoclonal antibody; Dako, Glostrup, Denmark), nephrin (anti-rabbit nephrin polyclonal antibody; kindly provided by Dr. Lawrence B. Holzman, University of Pennsylvania, Philadelphia, USA), synaptopodin (anti-mouse synaptopodin monoclonal antibody; Progen Biotechnik, Heidelberg, Germany), macrophages (anti-rat ED-1 monoclonal antibody; AbD Serotec, Raleigh, USA), proliferating cell nuclear antigen (anti-rat PCNA monoclonal antibody; Dako, Glostrup, Denmark), and lymphocytes (anti-rat CD43 monoclonal antibody; AbD Serotec, Raleigh, USA). Briefly, after incubation with the primary antibodies, the slides were subjected to a second reaction either with rat-adsorbed biotinylated anti-mouse IgG (Vector Labs, Burlingame, CA) or with biotinylated anti-rabbit IgG (Vector Labs). To complete the sandwich, sections were incubated with streptavidin-biotin-alkaline phosphatase complex (Dako, Glostrup, Denmark) for ED-1, and CD-43. Finally, the sections were incubated with a freshly prepared substrate consisting of naphtol-AS-MX-phosphate (Sigma Chemical Company) and fast red dye (Sigma Chemical Company). To evaluate the expression of WT1, nephrin and synaptopodin, the Novolink kit (Leica Novocastra, Newcastle Upon Tyne, United Kingdom) was used with peroxidase, protein, and post primary blocks, supplied by the kit, as the polymer and substrate.

Quantitative analysis of macrophages, lymphocytes and proliferative activity was carried out in a blinded fashion under 200x microscope magnification and expressed as cells/mm^2^. The podocyte marked area of the renal cortex was determined by histomorphometry using an image analysis system consisting of a light microscope (Eclipse E800; Nikon, Tokyo, Japan) coupled to a digital camera (Evolution; Media Cybernetics, Rockville, MD) with Q-Capture 2.95.0 graphic interface software (Quantitative Imaging, Surrey, BC, Canada). High-quality images (2048 × 1536-pixel buffer) were captured, and after calibration of the program settings, images were analyzed using Image ProPlus software (version 4.5.1; Media Cybernetics). The brightness and contrast of the images were adjusted after quantification to obtain better representation.

### Apoptosis assay

Apoptosis in kidney samples was evaluated in paraffin-embedded tissue with an *in situ* cell death detection kit (Roche, Penzberg, Germany). The terminal deoxynucleotidyltransferase mediated deoxyuridine triphosphate nick-end labeling-positive (TUNEL^+^) cells were quantified in 10 randomly fields per slide (×400 magnification) by an investigator who was blinded to the groups. To obtain the mean numbers of TUNEL^+^ cells in the kidney, all fields were evaluated and the mean counts per all kidney cells (obtained by staining with 4′-6-diamidino-2-phenylindole) were calculated as percentages.

### Real-time quantitative PCR

A quantitative real-time reverse transcription (RT) polymerase chain reaction (PCR) was performed to measure, in the kidney, the relative levels of Wilms’ tumor suppressor 1 (WT1), nephrin, podocin, synaptopodin, podocalyxin, IL-1β, TNF-α, IL-4, IL-10, and vascular endothelial growth fator (VEGF). Left kidneys were cut transversally in slices, collected in cryotubes, flash-frozen by immersion in liquid nitrogen, and stored at −80 °C. Total RNA was extracted with an RNeasy kit (QIAGEN, Valencia, CA), including a DNase-digestion step to exclude DNA contamination. RNA concentration was measured by spectrophotometry in a Nanodrop ND-2000. First-strand cDNA was synthesized from total RNA using a QuantiTect Reverse Transcription Kit (QIAGEN). Real-time PCR primers for the target gene were purchased from Invitrogen (Carlsbad, CA). Relative mRNA levels were measured with the BRYT Green system (Promega, Fitchburg, WI) using PCR Mastercycler Realplex Eppendorf (Eppendorf, Hamburg, Germany). All experiments were performed in triplicate. Real-time PCR conditions were as follows: 50 °C for 10 minutes, 95 °C for 5 minutes, 95 °C for 15 seconds, 60 °C for 30 seconds, and 72 °C for 30 seconds, with analysis of the fluorescence emission at 65 °C. Forty cycles were performed for each experiment. The relative expression of each gene was calculated as a ratio of the gene under study to the control gene, β-actin, and expressed as the fold change relative to the control (2^−ΔΔCt^). The PCR primers used are listed in Supplementary Table 4.

### Statistical analyses

Data were analyzed using GraphPad Prism 5 (GraphPad Software, La Jolla, CA). Selection of the appropriate statistical tests was based on the variance and the underlying distribution of the data. Global effects between groups were first assessed using one-way analysis of variance with Newman-Keuls correction for multiple comparisons, and the results are expressed as the mean ± SEM, with *p* < 0.05 considered statistically significant.

## Supplementary information

Supplementary information

## Data Availability

All data generated or analysed during this study are included in this published article and its Supplementary Information Files.
