# Supplementary material for: Mesenchymal Stromal Cells Induce Podocyte Protection in the Puromycin Injury Model
Source: Sci Rep. 2019 Dec 20;9:19604. doi: 10.1038/s41598-019-55284-7 (PMC6925195; doi:10.1038/s41598-019-55284-7)
Supplement: Supplementary file 1 — Supplementary information [file 41598_2019_55284_MOESM1_ESM.docx]

**Mesenchymal Stromal Cells Induce Podocyte Protection**

**in the Puromycin Injury Model**

**Felipe Mateus Ornellas^1^, Rodrigo J. Ramalho^2^, Camilla Fanelli^2^, Margoth Ramos Garnica^2^, Denise M.A.C. Malheiros^2^, Sabrina Vargas Martini^1^, Marcelo Marcos Morales^1^_,_Irene L. Noronha^2^***

^1^ Laboratory of Cellular and Molecular Physiology, Institute of Biophysics Carlos Chagas Filho, Federal University of Rio de Janeiro, Rio de Janeiro, Brazil

^2^ Laboratory of Cellular, Genetic, and Molecular Nephrology, Renal Division, University of São Paulo, São Paulo, Brazil

Felipe Mateus Ornellas and Rodrigo J. Ramalho contributed equally to this work

**Short title:** *Mesenchymal Stromal Cells Induce Podocyte Protection*

**Correspondence:*

Irene L. Noronha, MD, PhD

Laboratorio de Nefrologia Celular, Genetica e Molecular

Faculdade de Medicina – Universidade de São Paulo

Av. Dr. Arnaldo, 455, 4o andar, Lab 4304

São Paulo, CEP 01246-903, Brasil

Tel: +5511 30618403

Fax: +5511 30618361

Email: [irenenor@usp.br](mailto:irenenor@usp.br)

**SUPPLEMENTARY DATA**

**Table 1.** BUN and serum creatinine at days 0, 30 and 60

|  | **BUN**  (mg/dL) | | | **serum Creatinine**  (mg/dL) | | |
| --- | --- | --- | --- | --- | --- | --- |
| **Day** | **0** | **30** | **60** | **0** | **30** | **60** |
| **Control** | 19±2 | 34±3 | 25±1 | 0.33±0.1 | 0.48±0.1 | 0.28±0.1 |
| **PAN** | 19±1 | 73±5^*,#,^**^‡^** | 49±5^*,†,^**^‡^** | 0.34±0.1 | 1.1±0.2^*,#,^**^‡^** | 0.65±0.1^*,†,^**^‡^** |
| **PAN + mSC** | 17±2 | 69±13^*,#,§^ | 34±6 | 0.31±0.1 | 0.96±0.1^*,#,§^ | 0.46±0.1 |

^*^ p<0.05 vs. Control at Day 0

# p<0.05 vs Control at Day 30

† p<0.05 vs. Control at Day 60

**‡ **p<0.05 vs PAN at Day 0

**§** p<0.05 vs PAN+mSC at Day 0

**Table 2.** Comparative analysis of podocyte markers after 30 and 60 days in the different groups. (A) The expression levels of WT1, nephrin, podocin, synaptopodinandpodocalyxin were analyzed in renal tissue by real time RT-PCR. Data are expressed as the mean ± SEM

| **Parameters**  (Relative mRNA) | **WT1** | | **NEPHRIN** | | **PODOCIN** | | **SYNAPTOPODIN** | | | **PODOCALYXIN** | | |
| --- | --- | --- | --- | --- | --- | --- | --- | --- | --- | --- | --- | --- |
| **Day** | 30 | 60 | 30 | 60 | 30 | 66  60 | 30 | 60 | 30 | | 60 |  |
| **Control** | 1.0±0.32 | 1.0±0.13 | 1.0±0.11 | 1.0±0.15 | 1.0±0.10 | 1.0±0.24 | 1.0±0.03 | 1.0±0.21 | 1.0±0.13 | | 1.0±0.16 |  |
| **PAN** | 0.09±0.18* | 0.14±0.25* | 0.47±0.22 | 0.10±0.2* | 0.17±0.2* | 0.10±0.28* | 0.33±0.17* | 0.28±0.36 | 0.72±0.12 | | 0.53±0.27 |  |
| **PAN+mSC** | 0.42±0.21 | 0.86±0.23^#^ | 0.58±0.2 | 0.32±0.31 | 0.76±0.23 | 0.83±0.29 | 0.79±0.21 | 0.87±0.33 | 0.82±0.42 | | 0.91±0.27 |  |

*p<0.05 vs. Control; ^#^p<0.05 vs. PAN group.

**Table 3.** Comparative analysis of inflammatory cytokines and VEGF after 30 and 60 days in the different groups. (A) The expression levels of TNF-α, IL-1β, IL-6, IL-4andIL-10 were analyzed in renal tissue by real time RT-PCR. Data are expressed as the mean ± SEM.

| **Parameters**  (Relative mRNA) | **IL-1β** | | | **TNF**-α | | | **IL-4** | | **IL-10** | | **VEGF** | |
| --- | --- | --- | --- | --- | --- | --- | --- | --- | --- | --- | --- | --- |
| **Day** | **30** | **60** | | **30** | | **60** | **30** | **60** | **30** | **60** | **30** | **60** |
| **Control** | 1.0±0.17 | | 1.0±0.22 | | 1.0±0.22 | 1.0±0.37 | 1.0±0.14 | 1.0±0.2 | 1.0±0.16 | 1.0±0.21 | 1.0±0.28 | 01.0±0.13 |
| **PAN** | 1.58±0.19 | | 3.53±0.21 | | 1.28±0.27 | 2.31±0.2* | 0.12±0.18* | 0.18±0.11* | 1.47±0.19 | 3.77±0.19* | 0.6±0.15 | 00.39±0.2* |
| **PAN+mSC** | 1.11±0.2 | | 2.44±0.32 | | 1.05±0.45 | 1.44±0.39^#^ | 0.09±0.03* | 1.34±0.22^#^ | 2.1±0.21* | 5.25±0.32*^,#^ | 0.85±0.18 | 0.99±0.17^#^ |

*p<0.05 vs. Control; ^#^p<0.05 vs. PAN group.

**Table 4.** Primer sequences used for qRT-PCR

| **Gene** | **Sense** | **Anti-sense** |
| --- | --- | --- |
| ß-actin | 5′ AGGAGTACGATGAGTCCGGCCC 3′ | 5′ GCAGCTCAGTAACAGTCCGCCT 3′ |
| Nephrin | 5′ TAATGTGTCTGCGGCCCAG 3′ | 5′ TGTTGGTGTGGTCAGAGCCA 3′ |
| Podocin | 5′ GACGCTGTCTGCTACTACCGCAT 3′ | 5′ CGATGTGCCAAGAGGCGCTTC 3′ |
| Synaptopodin | 5′ GAATCTATGGCACGCCGAGGCAG 3′ | 5′ TGCCTCCGCTTCTCATCAGCTGT 3′ |
| Podocalyxin | 5′ CTGCATGGCATCCTTCCTGCTCC 3′ | 5′ TCCTCTGGGAGATCCGCTGGTG 3′ |
| IL-1β | 5′ CCTTGTGCAAGTGTCTGAAGCAGC 3′ | 5′ GCCACAGCTTCTCCACAGCCA 3′ |
| TNF-α | 5′ TGGCCCAGACCCTCACACTCA3′ | 5′GGCTCAGCCACTCCAGCTGC 3′ |
| IL-4 | 5′ TGGGTCTCAGCCCCCACCTT 3′ | 5′ TCCGTGGATACCGTTCCCGGT 3′ |
| IL-10 | 5′ TACCTGGTAGAAGTGATGCCCCAGG 3′ | 5′ CACAGGGGAGAAATCGATGACAGCG 3′ |
| VEGF | 5′ ACTGTGAGCCTTGTTCAGAGCGG 3′ | 5′ TCAAGCTGCCTCGCCTTGCA 3′ |
| WT-1 | 5′ GGACCCTTCGGTCCTCCCCC 3′ | 5′ GCTGGCTCTCCAGGCAGCTG 3′ |

**Figure 1.** The Masson Trichrome technique was employed to evaluate the percentage of interstitial fibrosis in renal specimens. PAN animals exhibited significantly higher interstitial fibrosis. mSC treatment attenuated this parameter at day 30, but not at day 60.

* p<0.05 vs Control

^#^ p<0.05 vs PAN

**Figure 2.** Illustrative microphotographs and quantification of renal apoptosis achieved by an *in situ* TUNEL fluorescence staining kit. No apoptosis was observed in the Control group at day 30 **(A)** and 60 **(D)**. PAN animals showed an increased number of apoptotic cells (white arrows) at both days 30 **(B)** and 60 **(E)**. Animals treated with mSC showed reduced renal apoptosis at both days 30 **(C)** and 60 **(F)**. The quantification of the percentage of apoptotic cells in all the experimental groups is represented in figure **(G).** Renal apoptosis was significantly higher in the PAN group at days 30 and 60, when compared with controls. The occurrence of apoptosis in PAN rats treated with mSC treatment was significantly lower after 60 days.

**PAN**

**PAN+mSC**

**Control**


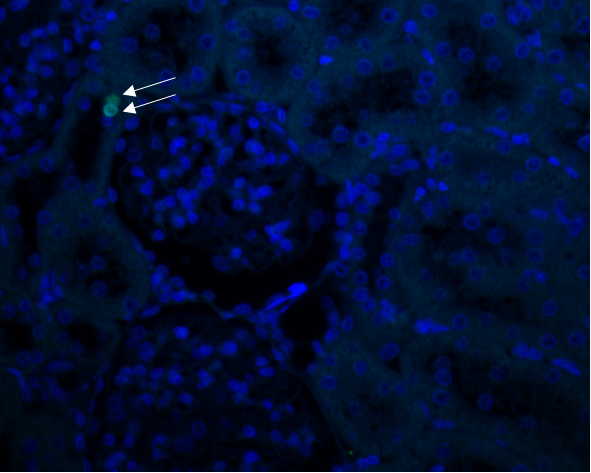

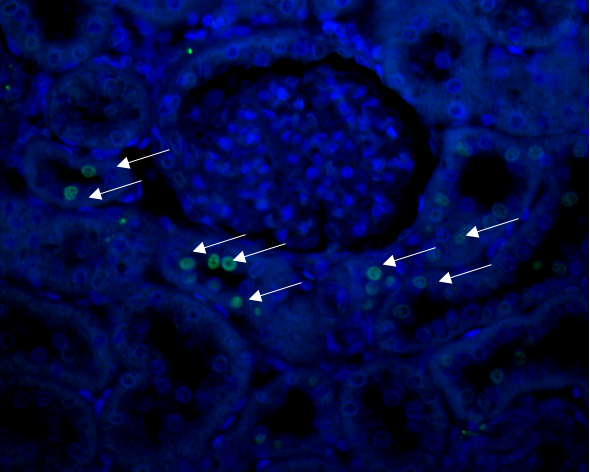

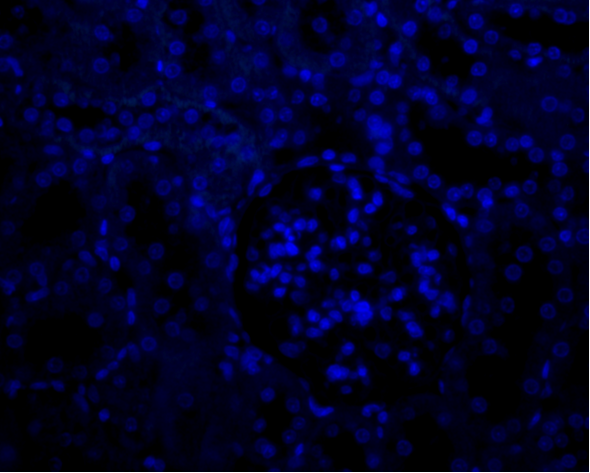


**A**

**B**

**C**

**D**

**E**

**F**

**Day 30**

**
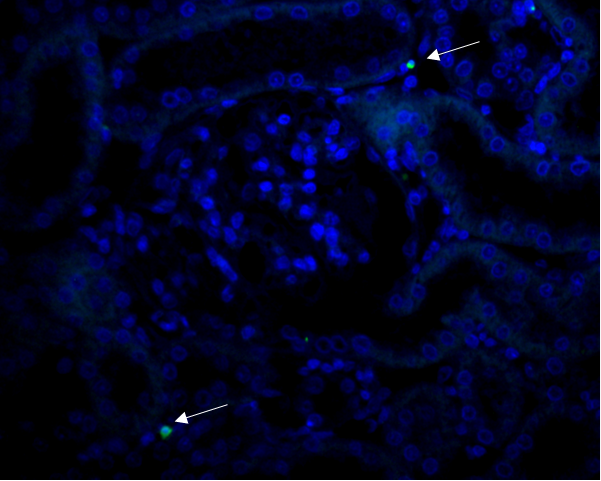
**
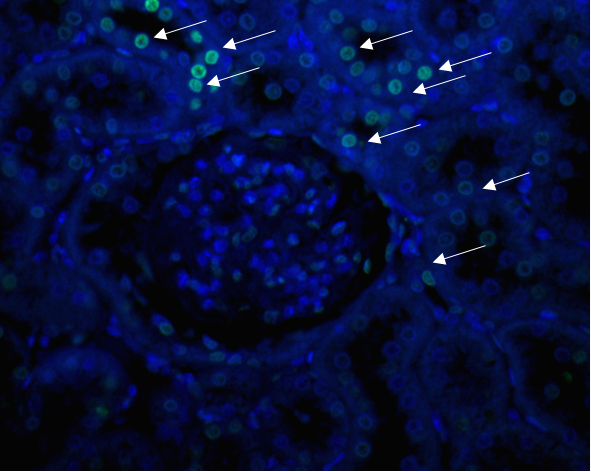

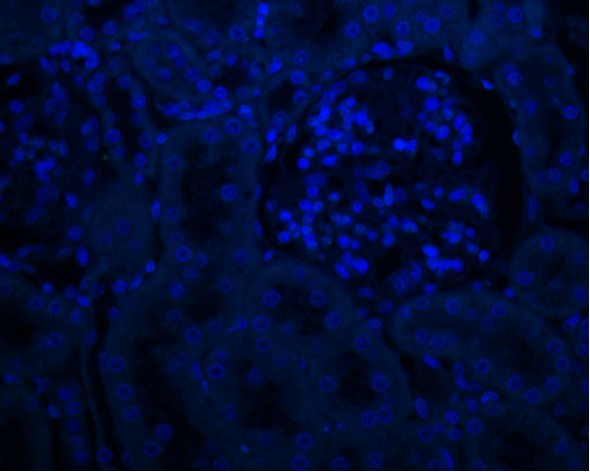


**Day 60**

**G**

* p<0.05 vs Control

**Figure 3.** The presence of tubular atrophy **(A)** and tubular casts **(B)** was analyzed in 25 consecutive microscopic fields of PAS-stained kidney sections of all experimental groups, under 200x magnification. Control animals did not present tubular atrophy. PAN rats showed modest tubular atrophy at day 30, which drastically increased at day 60. Treatment with mSC did not significantly improve tubular atrophy **(A)**. Tubular casts were not detected in control rats. Tubular hyaline casts were prominent in PAN animals at both 30 and 60 days, in parallel with the findings of proteinuria and albuminuria. mSC treatment numerically reduced the presence of tubular casts in groups PAN+mSC at both 30 and 60 days **(B)**.

**A**

* p<0.05 vs Control

**B**

**Figure 4.** Glomerular volume was analyzed in PAS-stained renal sections through a point-counting technique. PAN animals presented numerically increased glomerular volume at day 60, when compared to Control animals. However, no differences were observed between PAN and PAN+mSC regarding this parameter
